# Correction: Spatial cell fate manipulation of human pluripotent stem cells by controlling the microenvironment using photocurable hydrogel

**DOI:** 10.1242/dev.205058

**Published:** 2025-07-24

**Authors:** Zhe Wang, Akira Numada, Fumi Wagai, Yusuke Oda, Masatoshi Ohgushi, Koichiro Maki, Taiji Adachi, Mototsugu Eiraku

There was an error in Development (2024) **151**, dev201621 (doi:10.1242/dev.201621).

In Fig. S7C, the T channel immunostaining image was inadvertently duplicated in two panels. The corrected and original figures are shown below.

Fig. S7 (corrected):

**Figure:**
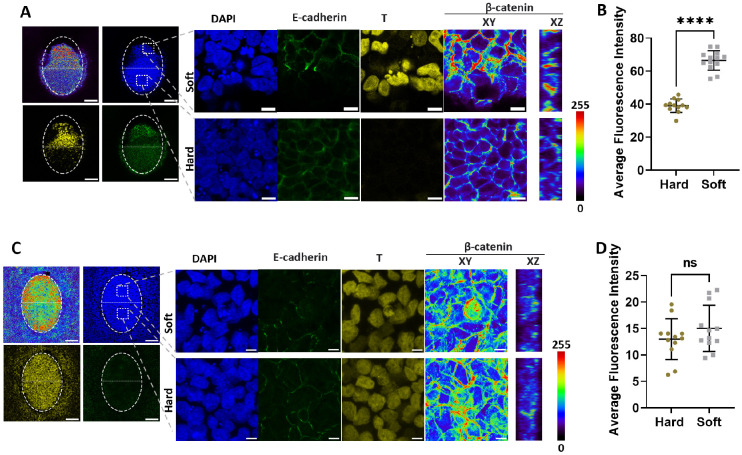


Fig. S7 (original):

**Figure:**
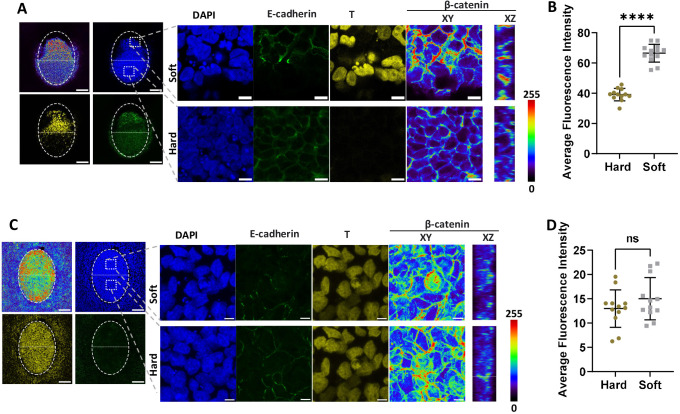


The online PDF of the supplementary file has been corrected.

The authors apologise to readers for this error, which does not impact the results and conclusions of the paper.

