# Supplementary material for: Spatial cell fate manipulation of human pluripotent stem cells by controlling the microenvironment using photocurable hydrogel
Source: Development. 2024 Mar 21;151(6):dev201621. doi: 10.1242/dev.201621 (PMC11006369; doi:10.1242/dev.201621)
Supplement: Supplementary information [file develop-151-201621-s1.pdf]

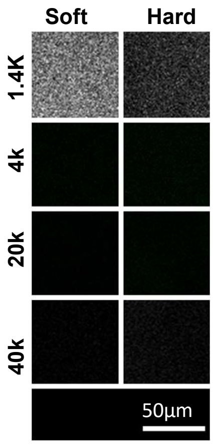

Fig. S1. Fluorescent Diffusion Experiment

Hydrogels were incubated with fluorescent molecules and then observed using laser confocal microscopy to assess the diffusion of fluorescent molecules inside the gel.

Scale bars: 50 µm.

Development • Supplementary information

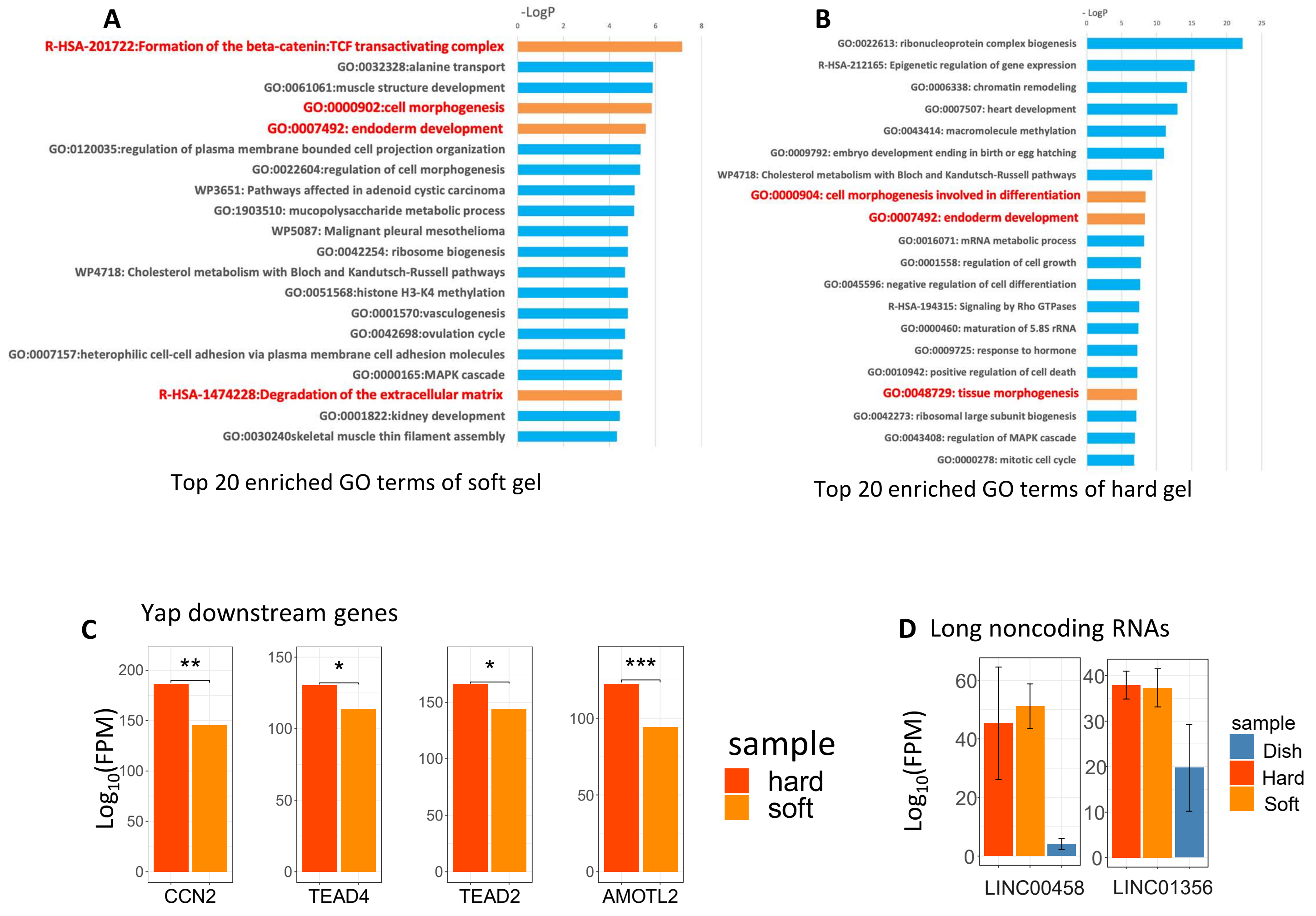

Fig. S2. (A) Top 20 Gene Ontology (GO) Terms (Soft vs. Dish): The top 20 Gene Ontology (GO) terms highlighting the categories of up-regulated genes in the cells on soft gel compared to those on dishes. Terms shown in red are involved in development. (B) Top 20 Gene Ontology (GO) Terms (Hard vs. Dish): The top 20 Gene Ontology (GO) terms highlighting the categories of up-regulated genes in the cells on hard gel compared to those on dishes. Terms shown in red are involved in development. (C) YAP down steam genes Expression Based on RNA-seq: Expression levels of selected genes based on RNA-seq of hPSCs cultured on soft/hard gel. Expression level is shown as TPM (Transcripts Per Kilobase Million). (D) Long non coding RNAs Expression Based on RNA-seq: Expression level is shown as TPM (Transcripts Per Kilobase Million).

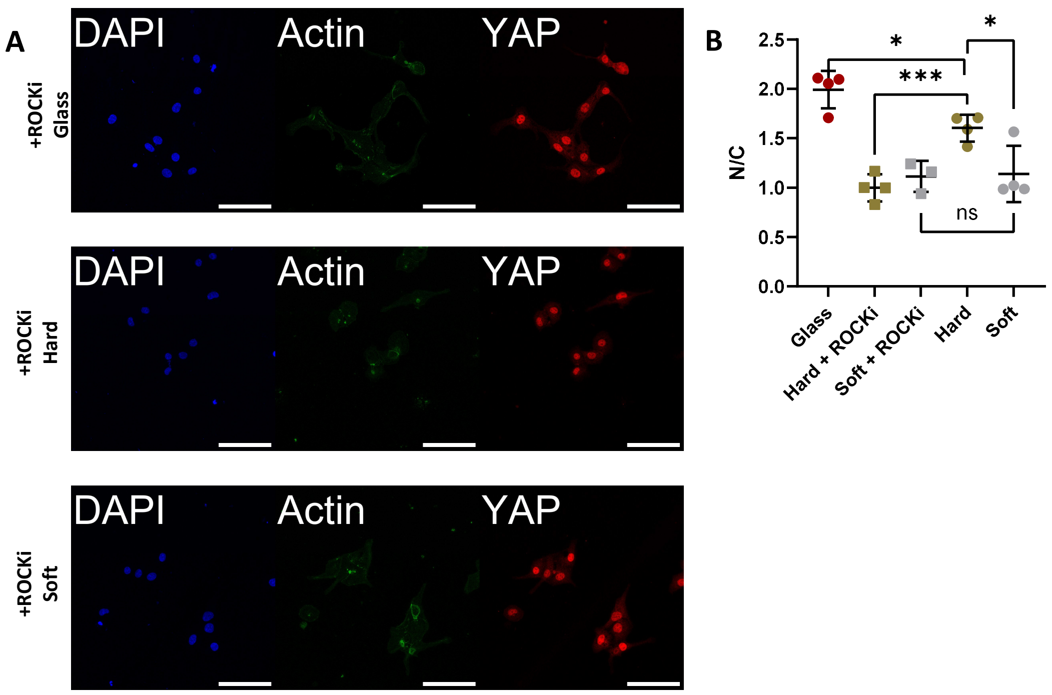

Fig. S3. Immunofluorescent (IF) Staining and YAP Quantification. (A) IF Staining of YAP: Immunofluorescent staining of YAP in cells cultured on a soft/hard gel or glass with 10μM Y-27632. Scale bars: 100 μm. (B) Quantification of YAP Nuclear Localization: YAP nuclear localization was quantified using the Nuclear/Cytoplasmic fluorescent intensity ratio (N/C).

Data obtained from at least 3 biological replicates. Each dot represents a quantification. Mean values, indicated by black lines, and error bars represent standard deviation. Statistical significance was determined using an unpaired, two-tailed t-test: ns - Not significant, *** P < 0.001, * P < 0.1.

Development • Supplementary information

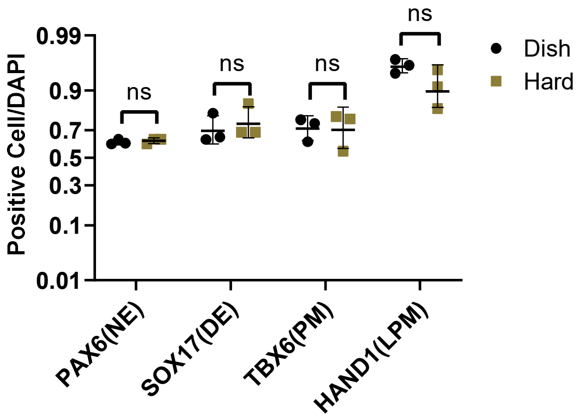

Fig. S4. Differentiation Efficiency Quantification: Positive cell ratio of PAX6 (neural ectoderm), SOX17 (definitive endoderm), TBX6 (Paraxial mesoderm), HAND1 (lateral plate mesoderm).

Data were obtained from at least 3 biological replicates Black lines represent median values. ns: Not significant. Error bars represent standard deviation.

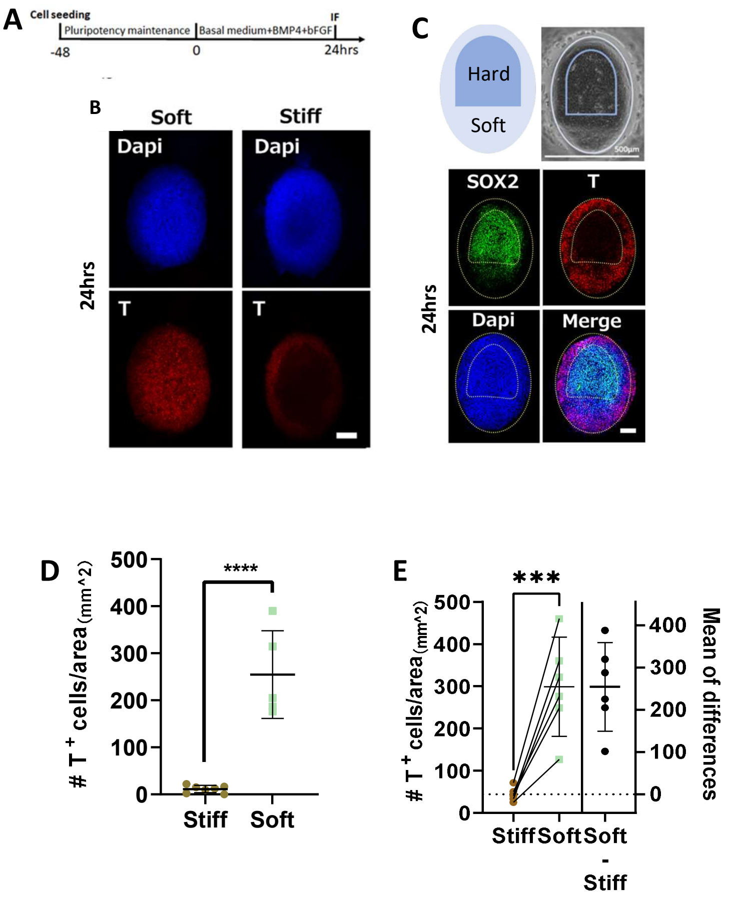

Fig. S5. Manipulating Stem Cell Fate by Adjusting Local Stiffness of the Hydrogel

(A) Differentiation Timetable for Mesoderm Differentiation (B) Immunofluorescence (IF) of 24-hour Mesoderm Differentiation on Soft and Hard Gels Scale bars: 100 μm. (C) IF of 24-hour Mesoderm Differentiation on Gel with Locally Different Stiffness Scale bars: 500 μm in bright filde,100 μm in IF image. (D) Quantification of T^+ Number/Area on Soft and Hard Gels (*n*=5) (E) Quantification of T^+ Number/Area on Each Gel with Locally Different Stiffness (*n*=6). Dashed line-connected dots represent soft and hard parts from the same patterned gel.

Data were obtained from at least 3 biological replicates. Black lines indicating median values. Error bars represent standard deviation. Statistical significance: **** P < 0.0001, *** P < 0.001 (unpaired, two-tailed t-test).

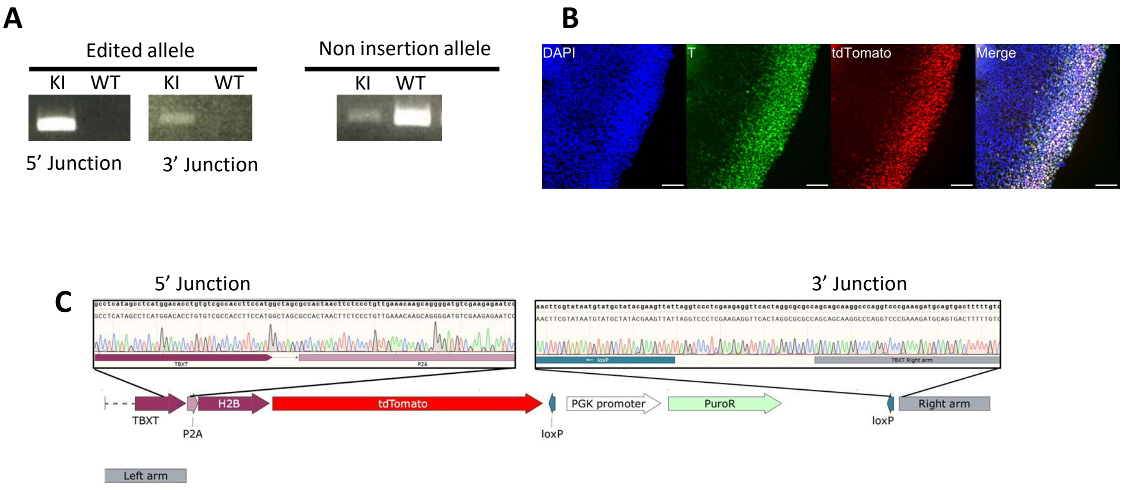

Fig. S6. Verification of Knock-in Reporter T-H2B-tdTomato (A) Genomic PCR of T-H2B-tdTomato: Electrophoresis showing successful heterogeneous integration into the genome. (B) Immunofluorescence (IF) of T-H2B-tdTomato: Differentiated with 10μM CHIR for 24 hours, showing colocalization of tdTomato and T expression. Scale bars: 100 μm. (C) Sanger Sequencing of Junction of Integrated Fragment

Development • Supplementary information

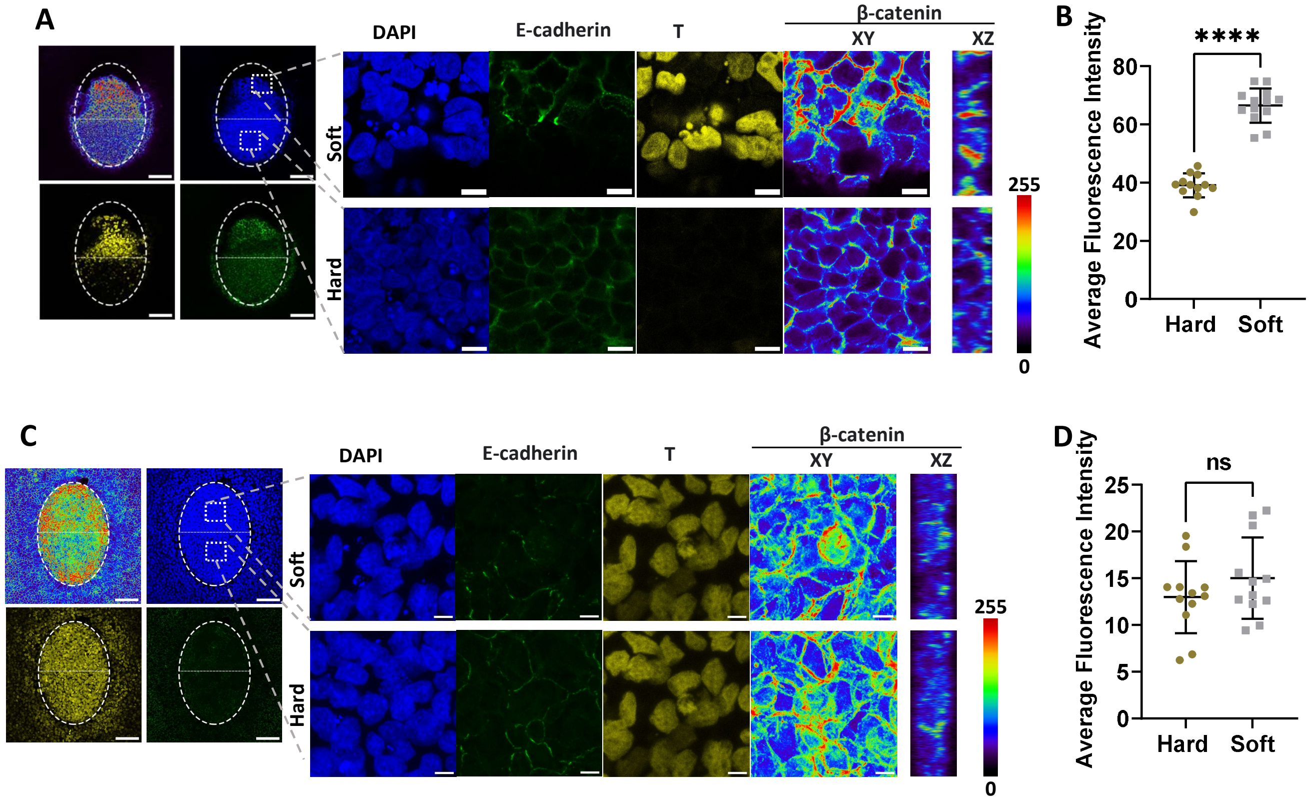

Fig. S7. Stem Cell Fate Control Can Be Achieved with BMP/bFGF but Not CHIR (A) IF Staining (Differentiated with BMP/bFGF): Immunofluorescent staining of hPSCs on a partially soft gel pattern using pulse differentiation, as shown in Fig. 5C. The white dotted oval line indicates the entire gel pattern, with the upper half being soft and the lower half being hard. The white box indicates a magnified view of the indicated region. The β-catenin channel is shown in XY and XZ section views as a heat map, with colors representing fluorescent intensity. Scale bars: 100 μm in the whole gel view and 10 μm in the magnified view. (B) Quantification of β-catenin in BMP/bFGF Differentiation: Quantification of β-catenin fluorescent intensity based on Z sections of IF staining in BMP/ bFGF differentiation on a partially soft gel.(*n*=12) (C) IF Staining (Differentiated with CHIR): Immunofluorescent staining of hPSCs on a partially soft gel pattern using pulsed differentiation with CHIR. The white dotted oval line indicates the entire gel pattern, with the upper half being soft and the lower half being hard. The white box indicates a magnified view of the indicated region. The β-catenin channel is shown in XY and XZ section views as a heat map, with colors representing fluorescent intensity. Scale bars: 100 μm in the whole gel view and 10 μm in the magnified view. (D) Quantification of β-catenin in CHIR Differentiation: Quantification of β-catenin fluorescent intensity based on Z sections of IF staining in CHIR differentiation on a partially soft gel (*n*=12).

Data were obtained from at least 3 biological replicates. Each dot represents an individual measurement. The black line represents the mean value. Statistical significance: ns - Not significant **** P < 0.0001 (unpaired, two-tailed t-test). Error bars represent standard deviation.

Table S1. List of oligonucleotides

Amplicon	Forward	Reverse	Ref
Oct4	CCTGAAGCAGAAGAGGATCACC	AAAGCGGCAGATGGTCGTTTGG	*Origene, HP206340*
PAX6	CTGAGGAATCAGAGAAGACAGGC	ATGGAGCCAGATGTGAAGGAGG	*Origene, HP225517*
OTX2	GGAAGCACTGTTTGCCAAGACC	CTGTTGTTGGCGGCACTTAGCT	*Origene, HP214209*
SOX1	GAGTGGAAGGTCATGTCCGAGG	CCTTCTTGAGCAGCGTCTTGGT	*Origene, HP209017*
PBGD	GGAGCCATGTCTGGTAACGG	CCACGCGAATCACTCTCATCT	*Kyle M. Loh et al., 2014*
FOXA1	AAGGCATACGAACAGGCACTG	TACACACCTTGGTAGTACGCC	*Ang et al., 1993; Sasaki and Hogan, 1993*
FOXA2	GGGAGCGGTGAAGATGGA	TCATGTTGCTCACGGAGGAGTA	*Ang et al., 1993; Sasaki and Hogan, 1993*
Hhex	CACCCGACGCCCTTTTACAT	GAAGGCTGGATGGATCGGC	*Thomas et al., 1998*
SOX17	CGCACGGAATTTGAACAGTA	GGATCAGGGACCTGTCACAC	*Kanai-Azuma et al., 2002*
TBX6	AAGTACCAACCCCGCATACA	TAGGCTGTCACGGAGATGAA	*Kyle M. Loh et al., 2016*
MSGN1	CGGAATTACCTGCCACCTGT	GGTCTGTGAGTTCCCCGATG	*Kyle M. Loh et al., 2016*
CDX2	GGGCTCTCTGAGAGGCAGGT	CCTTTGCTCTGCGGTTCTG	*Kyle M. Loh et al., 2016*
HAND1	GTGCGTCCTTTAATCCTCTTC	GTGAGAGCAAGCGGAAAAG	*Kyle M. Loh et al., 2016*
ISL1	AGATTATATCAGGTTGTACGGGATCA	ACACAGCGGAAACACTCGAT	*Kyle M. Loh et al., 2016*
NKX2.5	CAAGTGTGCGTCTGCCTTT	CAGCTCTTTCTTTTCGGCTCTA	*Kyle M. Loh et al., 2016*
Amplify H2B-tdTomato insert	tcccgggccaatgccagagccagcgaagt	gatcggaattcttacttgtacagctcgtccatgccg	Not applicable
Linearize T-2A-EGFP-PGK-Puro	tgtacaagtaagaattccgatcatattcaataaccct	gctctggcattggcccgggattctcttcgac	Not applicable
Genomic PCR of left junction	catcttcctgatgattttgtttcttattaatagatacgacaa	gctctggcattggcccgggattctcttcgac	Not applicable
Genomic PCR of right junction	tgtacaagtaagaattccgatcatattcaataaccct	ctgtcctcaactatgattttattctgtccttaacag	Not applicable
Sequencing left junction	tgagctctgaatatgtgaataatcttttcagtcatct	gctctggcattggcccgggattctcttcgac	Not applicable
Sequencing right junction	tgtacaagtaagaattccgatcatattcaataaccct	agttatagtttaacaacacaagagattagctacatatgc	Not applicable

Development • Supplementary information

Table S2. List of antibodies

Target	Manufacturer	Host animal	Dilution
T	R&D, AF2085	Goat	500
SOX2	Abcam, ab79351	Mouse	100
TBX6	R&D, AF4744	Goat	500
T	Abcam, ab20680	Rabbit	100
SOX17	R&D, AF1924	Goat	40
PAX6	R&D, AF8150	Mouse	1000
SOX1	Cell signaling, #4194	Rabbit	500
CDX2	Abcam, ab76541	Rabbit	500
HAND1	R&D, AF3168	Goat	200
NANOG	Abcam, #4893	Goat	500
OCT3/4	Abcam, ab181557	Rabbit	500
Alexa Fluor™ 546 Phalloidin	Invitrogen, A22283	Not applicable	500
Alexa Fluor™ 647 Phalloidin	Invitrogen, A22287	Not applicable	500
E-cadherin	Takara, ECCD-2	Mouse	200
β-Catenin	Sigma, C2206	Rabbit	1000
YAP	Santa Cruz Biotechnology, sc-101199	Mouse	200

Table S3. List of fluorescent molecules

	Manufacturer	Molecular weight	Concentration
Alexa Fluor™ 488 Phalloidin	Invitrogen, A22283	1.4kDa	100ng/ml
FITC-dextran 4	TdB Labs, FD4	4kDa	100ng/ml
FITC-dextran 20	TdB Labs, FD20	20kDa	100ng/ml
FITC-dextran 40	TdB Labs, FD40	40kDa	100ng/ml

Development • Supplementary information

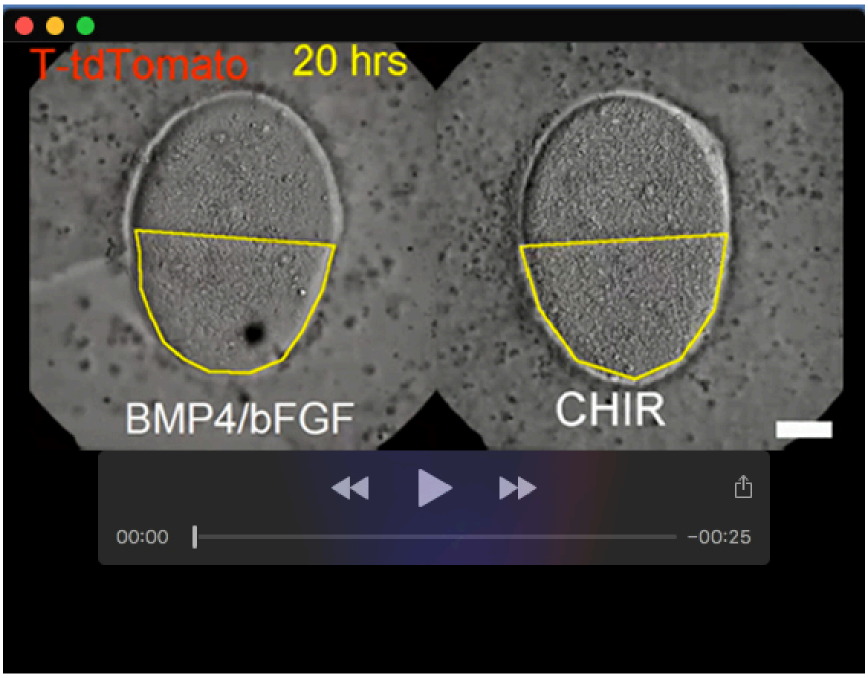

Movie 1. Spatial Cell Fate Control - Live imaging of T-H2B-tdTomato cells undergoing differentiation on a partially soft gel using BMP/bFGF or CHIR. The tdTomato signal is represented in red. The soft area of the gel is highlighted with a yellow circle. The live imaging was recorded at a 1-hour per frame rate.

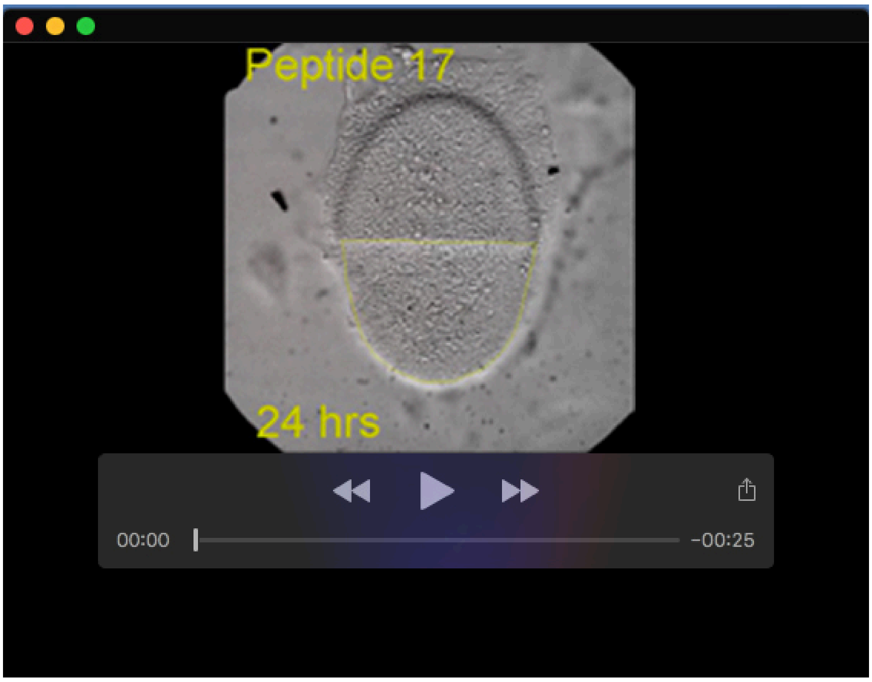

Movie 2. YAP Inhibition Disrupts Stem Cell Fate Control: Live Imaging of T-H2B-tdTomato Cells Undergoing Differentiation on a Partially Soft Gel using BMP/bFGF in the Presence of 50nM Peptide 17. The tdTomato signal is represented in red, while the soft area of the gel is highlighted with a yellow circle. The live imaging was recorded at a rate of one frame per hour.

Development • Supplementary information
