# Supplementary material for: Spatial cell fate manipulation of human pluripotent stem cells by controlling the microenvironment using photocurable hydrogel
Source: Development. 2024 Mar 21;151(6):dev201621. doi: 10.1242/dev.201621 (PMC11006369; doi:10.1242/dev.201621)
Supplement: Supplementary file 1 [file develop-151-201621_review_history.pdf]

Spatial cell fate manipulation of human pluripotent stem cells by controlling the microenvironment using photocurable hydrogel

Zhe Wang, Akira Numada, Fumi Wagai, Yusuke Oda, Masatoshi Ohgushi, Koichiro Maki, Taiji Adachi and Mototsugu Eiraku

DOI: 10.1242/dev.201621

Editor: Matthias Lutolf

Review timeline

Original submission:	13 April 2023
Editorial decision:	18 May 2023
First revision received:	25 October 2023
Editorial decision:	15 November 2023
Second revision received:	3 December 2023
Editorial decision:	3 January 2024
Third revision received:	24 January 2024
Accepted:	21 February 2024

Original submission

First decision letter

MS ID#: DEVELOP/2023/201621

MS TITLE: Spatial cell fate manipulation of human pluripotent stem cells by controlling the microenvironment using photocurable hydrogel

AUTHORS: Zhe Wang, Akira Numada, Yusuke Oda, Fumi Wagai, Masatoshi Ohgushi, and Mototsugu Eiraku

I have now received all the referees' reports on the above manuscript, and have reached a decision. The referees' comments are appended below, or you can access them online: please go to BenchPress and click on the 'Manuscripts with Decisions' queue in the Author Area.

As you will see, the referees express considerable interest in your work, but have some significant criticisms and recommend a substantial revision of your manuscript before we can consider publication. If you are able to revise the manuscript along the lines suggested, which may involve further experiments, I will be happy receive a revised version of the manuscript. Your revised paper will be re-reviewed by one or more of the original referees, and acceptance of your manuscript will depend on your addressing satisfactorily the reviewers' major concerns. Please also note that Development will normally permit only one round of major revision. If it would be helpful, you are welcome to contact us to discuss your revision in greater detail. Please send us a point-by-point response indicating your plans for addressing the referee's comments, and we will look over this and provide further guidance.

Please attend to all of the reviewers' comments and ensure that you clearly highlight all changes made in the revised manuscript. Please avoid using 'Tracked changes' in Word files as these are lost in PDF conversion. I should be grateful if you would also provide a point-by-point response detailing how you have dealt with the points raised by the reviewers in the 'Response to Reviewers' box. If you do not agree with any of their criticisms or suggestions please explain clearly why this is so.

© 2024. Published by The Company of Biologists under the terms of the Creative Commons Attribution License (https://creativecommons.org/licenses/by/4.0/).

Reviewer 1

Advance summary and potential significance to field

The paper by Eiraku and colleagues under consideration at Development describes a hydrogel platform where protein conjugation and mechanics can be tuned and are demonstrated to influence pluripotent stem cell (PSC) differentiation. The platform may prove of interest as a model system to study development, since they demonstrate control of stiffness and show some preliminary demonstrations of forming patterned regions. The demonstration of some signalling differences between gels and plastic, and between soft and stiff gels, is potentially interesting but there is a lack of mechanistic detail. Furthermore, the rationale for how the observed mechanics-signaling relationships relate to development and morphogenesis is lacking in the article.

Comments for the author

The following points should also be considered:
1. Does changing the stiffness of the gel influence the presentation/density of adhesion protein at the interface? This difference could also influence the changes in lineage specification observed between the “soft” and “stiff” regions. The authors need to (1) demonstrate successful conjugation of protein and (2) show how the laminin conjugation changes from a soft to a stiff hydrogel. If the density changes from soft to stiff, this needs to be discussed as a potential reason for the differences in signalling (like the NODAL results).

2. Why did the authors select a “hard” hydrogel (~100 kPa) for their initial PSC pluripotency studies? A recent study using polyacrylamide demonstrated some loss of pluripotency and gain of mes-endodermal identity for hPSCs cultured under growth conditions (Srivastava et al., Adv. Sci., 2023). Indeed, this recent study found similar trends in endoderm and mesoderm differentiation on account of stiffness; however, using a smaller range of stiffness. This paper should be discussed in the context of the authors results.

3. The influence of “softness” on NODAL-related signals is interesting, albeit entirely observational as it stands. The authors discuss axis specification and symmetry breaking in their GO analysis. Is there something about a softer substrates that could be discouraging pluripotency and favouring these pathways? The authors speculate that the softer gel has a larger meshsize which could enhance TGFb (and other) signals. However, without evidence to show enhanced paracrine signals in these regions, this is highly speculative. Are there other pathways, e.g. mechanotransduction, which might similarly influence the same downstream effectors? Some additional mechanistic evidence here would be helpful.

4. There are numerous errors in the text and the manuscript could use a thorough re-read. For instance, on the bottom of page 6 “…was extended to in h, cells” needs to be re-written.

Reviewer 2

Advance summary and potential significance to field

In this manuscript, Wang et al. developed a culture protocol for human pluripotent stem cells on 2D photo-crosslinkable polyvinyl alcohol (PVA), polyethylene glycol (PEG) gels functionalized with Laminin 511 to enable cell-matrix adhesion. Stiffness of PVA-PEG gel was patterned and tuned based on duration of exposure to ultraviolet light to either 20kPa (“soft”) or 100kPa (“hard”). hPSCs cultured on PVA-PEG hydrogels maintained pluripotency and showed proliferation dynamics that were similar to culture on standard 2D tissue culture plastic (polystyrene (PS)) dishes, and differentiated into neural ectoderm definitive endoderm and, paraxial and lateral mesoderm at an efficiency that was comparable to PS dishes.

The authors performed RNA-sequencing and found that hPSCs cultured on soft hydrogels showed upregulation of genes of the NODAL signaling pathway as compared to stiff substrates. Finally, using patterned gels with regions of soft and stiff modulus, the authors showed stiffness dependent

© 2024. Published by The Company of Biologists under the terms of the Creative Commons Attribution License (https://creativecommons.org/licenses/by/4.0/).

mesoderm differentiation with higher mesoderm differentiation on the soft regions. This manuscript demonstrates that spatial changes in stiffness can be used to pattern differentiation of hPSCs.

Comments for the author

Overall, Wang et al. demonstrate the use of hydrogels with tunable stiffness to direct differentiation in a spatially patterned manner. This proof-of-concept study highlights the value of mechanically tunable hydrogels for directing pluripotent stem cell fate. However, the study seems a bit preliminary in its current form and currently lacks sufficient characterization of the patterned hydrogels, analysis of the impact of stiffness on hPSC differentiation into all three germ layers and validation of involvement of mechanotransductive pathways. For publication in Development, a major revision would be necessary addressing the concerns listed below.

Major concerns:

1. Hydrogel characterization. The authors argue that their PVA-PEG hydrogel formulation can be used to spatially direct differentiation by spatially tuning hydrogel mechanical properties. However, this hydrogel system is not well characterized especially in case of patterned gels. AFM characterization of patterned gels should be performed to quantitatively confirm the spatial differences in stiffness. This is important to bolster the observations in Fig. 5. Further, the authors note in the Discussion section that differences in pore size might affect diffusion of activin and BMP4 to the basal surface of hPSCs and accordingly alter differentiation. Therefore, some characterization of hydrogel pore size should be performed for stiff and soft substrates (e.g. fluorescent molecule diffusion studies), and the potential role of pore size and not stiffness in mediating this effect should be discussed.

2. Impact of substrate stiffness on pluripotency maintenance, differentiation to neural ectoderm and definitive endoderm. In Fig. 2 and 3, pluripotency maintenance, proliferation rate and, differentiation to neural ectoderm and definitive endoderm were performed only on stiff substrates and compared to PS dishes. These experiments must be performed on soft substrates as well to test if pluripotency maintenance, proliferation and differentiation to other lineages are also influenced by substrate stiffness as reported previously (Shao et al., 2015). This would also help mitigate the concern about pore size noted in 1.

3. Mechanosensory pathways. While the authors performed RNA-sequencing for hPSCs cultured on soft and stiff substrates, it is unclear if any known mechanotransductive pathways are differentially regulated in soft and stiff substrates. At the very least, the authors should analyze existing RNA-sequencing data for differences in expression of genes involved in known mechanotransductive pathways. This is crucial to clarify if the observed differences in differentiation shown in Fig. 5 are due to mechanotransduction or due to other factors such as diffusion of morphogens. Additional analysis known mechanotransductive pathways via inhibition of cytoskeletal force generation or nuclear localization of YAP would be helpful.

Minor concerns:

1. In the abstract, the authors write “Biocompatible material that allows spatial control of the chemical and mechanical environment for hPSC culture has not yet been developed.” This is incorrect. While this biological result has not been demonstrated yet, biocompatible materials with spatial control have certainly been developed.

2. In Introduction, the authors state “2D culture systems are subject to mechanical constraints on tissue shape because cells are cultured on a rigid culture dish, whereas in 3D culture systems, induced tissues are not mechanically constrained and can freely change their shape.” This statement is factually incorrect.

3. All conclusions currently based on images should be supported by quantitative analyses with statistically significant differences.

© 2024. Published by The Company of Biologists under the terms of the Creative Commons Attribution License (https://creativecommons.org/licenses/by/4.0/).

4. What do the fluorescent lines in Fig. 1c indicate? Is distribution of Laminin 511 on hydrogels not homogeneous?

5. Scale bar is missing in Fig. 1c.

6. Figure captions lack description of bar plots and error bars, and in general do not have enough detail for the reader.

7. In the References list, several references are repeated.

8. Scale bars in Fig. 5 are not clearly defined. Are all Fig. 5 scale bars 100μm?

First revision

Author response to reviewers' comments

Below is a point-by-point response to each of the reviewer's comments:

Regarding the reviewer #1:
Comment 1st
Does changing the stiffness of the gel influence the presentation/density of adhesion protein at the interface? This difference could also influence the changes in lineage specification observed between the “soft” and “stiff” regions. The authors need to (1) demonstrate successful conjugation of protein and (2) show how the laminin conjugation changes from a soft to a stiff hydrogel. If the density changes from soft to stiff, this needs to be discussed as a potential reason for the differences in signalling (like the NODAL results).

Response 1: Concerning the stiffness and laminin density of the hydrogel, we conducted fluorescent protein binding experiments to indirectly measure the density by quantifying the fluorescent signal. The results show that soft and hard gels have a similar binding ability, and we also found it to be higher than in a glass-bottom dish, as shown in Figure 1. Laminin density is a critical point in our system, and we have added a part of the discussion how the high density of laminin affects cells in our system. In addition, previous fluorescent lines, likely due to sample drying, were absent in current experiments conducted in a buffer solution.

Comment 2nd
Why did the authors select a “hard” hydrogel (~100 kPa) for their initial PSC pluripotency studies? A recent study using polyacrylamide demonstrated some loss of pluripotency and gain of mes-endodermal identity for hPSCs cultured under growth conditions (Srivastava et al., Adv. Sci., 2023). Indeed, this recent study found similar trends in endoderm and mesoderm differentiation on account of stiffness; however, using a smaller range of stiffness. This paper should be discussed in the context of the authors results.

Response 2: We initially selected a "hard" hydrogel (~6kPa, as newly measured) to maintain a suitable environment for pluripotency maintenance, as softer environments have been shown to discourage pluripotency. Additionally, this stiffness range allowed us to generate well-defined gel patterns. We also conducted pluripotent marker staining on both soft and hard gels, and the cells maintained pluripotency within the observed time window (6 days). Regarding the enhancement of endoderm-related genes, we have discussed this based on further analysis of RNA-seq data. Endoderm and Nodal signaling are likely enhanced via the regulation of SMAD2/3 by LINC00458, as shown previously (DOI: 10.1126/sciadv.aay0264). We have included this reference in our paper and discussed it accordingly.

Comment 3rd
The influence of “softness” on NODAL-related signals is interesting, albeit entirely observational as it stands. The authors discuss axis specification and symmetry breaking in their GO analysis. Is there something about a softer substrate that could be discouraging pluripotency and favoring these

© 2024. Published by The Company of Biologists under the terms of the Creative Commons Attribution License (https://creativecommons.org/licenses/by/4.0/).

pathways? The authors speculate that the softer gel has a larger mesh size which could enhance TGFb (and other) signals. However, without evidence to show enhanced paracrine signals in these regions, this is highly speculative. Are there other pathways, e.g. mechanotransduction, which might similarly influence the same downstream effectors? Some additional mechanistic evidence here would be helpful.

Response 3: We analyzed the RNA-seq data and found no signs of pluripotency discouragement, as all major markers remained at similar levels to those in a glass dish. LINC00458, a stiffness-sensitive long noncoding RNA that regulates SMAD2/3 (DOI:10.1126/sciadv.aay0264), was found to be upregulated in our experiment, as well as SMAD2/3 downstream SMAD7. This likely explains the upregulation of endoderm-related genes in our system, similar to the previous study. We have included these findings in our paper's results and discussion sections.

Regarding the mesh size of the gel, we performed a fluorescent molecule diffusion experiment, and the results show that the mesh size of our gel is relatively small, not allowing molecules larger than 4 kDa to diffuse into the gel. This rules out the possibility of TGF-beta enhancement by the diffusion of proteins.
Thanks to the reviewer's advice, we found that YAP localization is indeed different in hard and soft gels, as shown in our new results. YAP is predominantly localized in the nucleus in hard gels and glass but not in soft gels. Interestingly, nuclear localization of YAP cannot be inhibited by using the Rho kinase inhibitor Y-27632. Since our hard gel is only ~6 kPa, we believe that the sustained nuclear localization of YAP is due to the high density of the adhesion protein laminin and its strong attachment to laminin 511. We have discussed this point in our paper. We also repeated ectoderm and endoderm differentiation on soft gel for comparison with hard gel and performed qPCR to assess the gene expression changes.

Comment 4th
There are numerous errors in the text and the manuscript could use a thorough re-read. For instance, on the bottom of page 6 "…was extended to in h, cells" needs to be re-written.

Response 4: We have carefully reviewed our manuscript to correct any minor errors, added scale bars, and improved the descriptions of the statistical analysis.

Regarding the reviewer #2:

Comment 1st
Hydrogel characterization. The authors argue that their PVA-PEG hydrogel formulation can be used to spatially direct differentiation by spatially tuning hydrogel mechanical properties. However, this hydrogel system is not well characterized especially in case of patterned gels. AFM characterization of patterned gels should be performed to quantitatively confirm the spatial differences in stiffness. This is important to bolster the observations in Fig. 5. Further, the authors note in the Discussion section that differences in pore size might affect diffusion of activin and BMP4 to the basal surface of hPSCs and accordingly alter differentiation. Therefore, some characterization of hydrogel pore size should be performed for stiff and soft substrates (e.g. fluorescent molecule diffusion studies), and the potential role of pore size and not stiffness in mediating this effect should be discussed.

Response 1: We appreciate your comment. According to your comment, we have performed AFM measurements on the patterned gel and confirmed a clear border of rigidity between soft gel and hard gel (Figure 5). We also conducted fluorescent molecule diffusion studies, as shown in Figure 1, and found that the mesh size of our gel is relatively small, not allowing molecules larger than 4 kDa to diffuse into the gel. This result rules out the possibility of TGF-beta signal enhancement by the diffusion of proteins.

Comment 2nd
.Impact of substrate stiffness on pluripotency maintenance, differentiation to neural ectoderm and definitive endoderm. In Fig. 2 and 3, pluripotency maintenance, proliferation rate and, differentiation to neural ectoderm and definitive endoderm were performed only on stiff substrates and compared to PS dishes. These experiments must be performed on soft substrates as well to test if pluripotency maintenance, proliferation and differentiation to other lineages are also influenced

© 2024. Published by The Company of Biologists under the terms of the Creative Commons Attribution License (https://creativecommons.org/licenses/by/4.0/).

by substrate stiffness as reported previously (Shao et al., 2015). This would also help mitigate the concern about pore size noted in 1.

Response 2: According to your suggestion, we have conducted immunostaining for pluripotency markers to assess the pluripotent state of the cells cultured on the soft gel and hard gel. Additionally, we have analyzed the cell cycle to evaluate proliferation rates on both soft and hard gels and dish. To further investigate differentiation potential, we have performed qPCR for differentiation into neural ectoderm and definitive endoderm on the soft gel, providing a direct comparison with the results obtained from the hard gel.

Comment 3rd
Mechanosensory pathways. While the authors performed RNA-sequencing for hPSCs cultured on soft and stiff substrates, it is unclear if any known mechanotransductive pathways are differentially regulated in soft and stiff substrates. At the very least, the authors should analyze existing RNA-sequencing data for differences in expression of genes involved in known mechanotransductive pathways. This is crucial to clarify if the observed differences in differentiation shown in Fig. 5 are due to mechanotransduction or due to other factors such as diffusion of morphogens. Additional analysis known mechanotransductive pathways via inhibition of cytoskeletal force generation or nuclear localization of YAP would be helpful.

Response 3: Thank you for your valuable comment. Reanalysis of RNAseq revealed that expression of several downstream genes of YAP, including CNN1 and CNN2, was significantly higher in hESCs cultured on hard gel than on soft gel (Supplementary Figure 2). We also performed YAP staining on both soft and hard gels to directly assess the localization and activity of YAP in response to substrate stiffness. Our results showed that YAP localized predominantly in the nucleus in hard gel and dish but not in soft gel. These observation is consistent with our hypothesis that substrate stiffness plays a role in YAP localization.

We also employed the cytoskeleton inhibitor Y-27632 to perturb these pathways experimentally. However, we found that nuclear-localized YAP was not inhibited on both glass and hard gel. This intriguing result may be attributed to the high attachment properties of laminin 511 or potentially to a mechanism of mechanosensing that is independent of ROCK signaling.

Furthermore, we revealed an enhancement in the formation of β-catenin on our soft gel, consistent with findings from another study where a soft substrate enhanced mesoderm differentiation (DOI:10.1016/j.stem.2016.06.018). To further investigate this, we performed live imaging, immunostaining, and perturbation of the Wnt/β-catenin signaling pathway. These additional experiments provided valuable insights, suggesting that the soft gel promotes Wnt signaling and, consequently, enhances mesoderm differentiation when treated with BMP4. The absence of nuclear YAP on the soft gel, coupled with the previously reported negative impact of YAP on Wnt signaling, supports our proposed mechanism: the soft gel promotes mesoderm differentiation via the loss of nuclear YAP and the enhancement of Wnt signaling. We have provided a detailed discussion of these findings in our results and discussion sections.

Minor concerns:

Comment 1:
In the abstract, the authors write, "Biocompatible material that allows spatial control of the chemical and mechanical environment for hPSC culture has not yet been developed." This is incorrect. While this biological result has not been demonstrated yet, biocompatible materials with spatial control have certainly been developed.

Response 1:
We have revised our statement to reflect this comment.

Comment 2:
In the Introduction, the authors state, "2D culture systems are subject to mechanical constraints on tissue shape because cells are cultured on a rigid culture dish, whereas in 3D culture systems,

© 2024. Published by The Company of Biologists under the terms of the Creative Commons Attribution License (https://creativecommons.org/licenses/by/4.0/).

induced tissues are not mechanically constrained and can freely change their shape." This statement is factually incorrect.

Response 2:
We have updated our statement to address this comment.

Comment 3:
All conclusions currently based on images should be supported by quantitative analyses with statistically significant differences.

Response 3:
We have added quantitative and statistical analysis for all images.

Comment 4:
What do the fluorescent lines in Fig. 1c indicate? Is distribution of Laminin 511 on hydrogels not homogeneous?

Response 4:
We have measured fluorescent intensity in the aqueous phase, and no lines were observed. We believe the lines in the previous picture were likely due to sample drying. However, in the picture acquired this time, we can observe some uneven distribution of fluorescent material, as seen in Fig 1G. This may suggest an uneven distribution of the hydrogel surface, which is unavoidable when using hydrogel.

Comment 5:
Scale bar is missing in Fig. 1c.

Response 5:
We have added scale bars to all images.

Comment 6:
Figure captions lack a description of bar plots and error bars, and in general do not have enough detail for the reader.

Response 6:
We have added descriptions for all figures.

Comment 7:
In the References list, several references are repeated.

Response 7:
We have resolved this issue.

Comment 8:
Scale bars in Fig. 5 are not clearly defined. Are all Fig. 5 scale bars 100μm?

Response 8:
We have added scale bars and descriptions for all images, including Fig. 5.

Second decision letter

MS ID#: DEVELOP/2023/201621

MS TITLE: Spatial cell fate manipulation of human pluripotent stem cells by controlling the microenvironment using photocurable hydrogel

© 2024. Published by The Company of Biologists under the terms of the Creative Commons Attribution License (https://creativecommons.org/licenses/by/4.0/).

AUTHORS: Zhe Wang, Akira Numada, Yusuke Oda, Fumi Wagai, Masatoshi Ohgushi, Koichiro Maki, Taiji Adachi, and Mototsugu Eiraku

I have now received all the referees reports on the above manuscript, and have reached a decision. The referees' comments are appended below, or you can access them online: please go to BenchPress and click on the 'Manuscripts with Decisions' queue in the Author Area.

The overall evaluation is positive and we would like to publish a revised manuscript in Development, provided that the referees' comments can be satisfactorily addressed. Please attend to all of the reviewers' comments in your revised manuscript and detail them in your point-by-point response. If you do not agree with any of their criticisms or suggestions explain clearly why this is so. If it would be helpful, you are welcome to contact us to discuss your revision in greater detail. Please send us a point-by-point response indicating your plans for addressing the referees' comments, and we will look over this and provide further guidance.

Reviewer 1

Advance summary and potential significance to field

This paper provides evidence that materials properties can spatially control differentiation in culture with involvement of mechanotransduction pathways and cytokine signaling. It also provides a platform to study germ layer specification.

Comments for the author

In the revised version of the manuscript the authors have performed several new experiments in response to the previous review. Overall the authors resolved some major aspects of my questions. The revision has led to some insight into mechanism where there appears to be involvement of Wnt/b-catenin signaling and YAP signaling. However there are still some issues that need to be addressed before the work may be considered further.

1) The finding that YAP shows cytoplasmic localization on the soft hydrogels is an interesting finding. They used the ROCK inhibitor and remarked how it was surprising that it didn't influence the YAP result. Here it seems that the soft material is catalyzing translocation which appears to be independent of ROCK-mediated contractility. It would have been interesting to see if an inhibitor of the YAP-TEAD network (i.e., peptide 17) would disrupt pluripotency on the hard gel, leading to T expression comparable to the soft gel.

2) The transciptome analysis of Figure 3 is only highlighting pluripotency and endoderm, but the remaining figures focus in on mesoderm. Is there a reason mesoderm differential expression was not highlighted here?

3) The authors have removed quantification from several figures and put them into supplement. The IF images should have accompanied quantification. In general, the figures could use some work as they are difficult to understand as presented. Each figure should tell a precise part of the overall story and I feel like they are now a bit disconnected in message. Having them separate from the legends at the end of the manuscript does not help interpretation.

4) The appearance of the T stain looks like the cells are differentiating away from the main body of the aggregate. This is a potentially interesting characteristic. Was this observed only for cells staining brachyury or was this also observed for the endoderm populations?

Minor:

- Figure 5 E –the label up-soft down hard is difficult to understand
- Figure 6 D –the axis label is cut off and it's difficult to follow what this graph is showing

Reviewer 2

© 2024. Published by The Company of Biologists under the terms of the Creative Commons Attribution License (https://creativecommons.org/licenses/by/4.0/).

Advance summary and potential significance to field

This manuscript is the first, to my knowledge, to show the spatially controlled patterning of hiPSCs using spatially controlled substrate stiffness.

Comments for the author

The authors have been partially responsive to the critiques that I raised. A few follow up comments:

1. The phrase "Biocompatible material that allows spatial control of the chemical and mechanical environment for hPSC culture has not yet been developed" should be removed from the abstracts. As I noted in my previous review, such biocompatible materials have been developed.

2. Quantitative analyses and statistics are still required (as noted in previous review) for Fig. 4 and 5.

3. For all statistical analyses, please include number of samples and biological replicates in the caption (and of course there should be at least two biological replicates)

3. Quantification of YAP should be of the nuclear: cytoplasmic ratio, not average intensity. Similarly, some quantification of b-catenenin localization to cell border would be helpful.

Second revision

Author response to reviewers' comments

Regarding the reviewer #1:
Comment 1st
The finding that YAP shows cytoplasmic localization on soft hydrogels is an interesting finding. They used the ROCK inhibitor and remarked how it was surprising that it didn’t influence the YAP result. Here it seems that the soft material is catalyzing translocation which appears to be independent of ROCK-mediated contractility. It would have been interesting to see if an inhibitor of the YAP-TEAD network (i.e., peptide 17) would disrupt pluripotency on the hard gel, leading to T expression comparable to the soft gel.
Response 1:
Thank you for recommending the YAP experiment. Initially, we quantified YAP localization using the mean intensity of nuclei and cytoplasm in the last revised manuscript. Upon reanalyzing our data, as suggested by the reviewer#2, and using total intensity, we found a decreased nuclear localization of YAP after ROCK inhibition. The lack of a significant change in YAP using mean intensity is likely attributed to the flattened and expanded morphology observed after ROCK inhibition.
Nevertheless, we have continued to investigate YAP inhibition in the spatial differentiation of stem cells. As suggested by the reviewer, we used peptide 17 and differentiated on soft/hard gel. Our newly performed live imaging in Movie2 demonstrates that treatment with Poptide 17 cancels differences in brachyury induction on gels of different stiffness.

Comment 2nd
The transciptome analysis of Figure 3 is only highlighting pluripotency and endoderm, but the remaining figures focus in on mesoderm. Is there a reason mesoderm differential expression was not highlighted here?
Response 2: The transcriptome analysis was conducted under maintenance conditions, revealing that cells retained pluripotency with no significant change in mesoderm gene expression. The remaining analysis was performed under differentiation conditions.

Moreover, during early lineage specification, both endoderm and mesoderm originate from the primitive streak. Distinguishing between endoderm and mesoderm at this early stage of

© 2024. Published by The Company of Biologists under the terms of the Creative Commons Attribution License (https://creativecommons.org/licenses/by/4.0/).

differentiation is challenging. Therefore, to maintain scientific accuracy, we utilized the term "mesendoderm" or "primitive streak".
Comment 3rd
The authors have removed quantification from several figures and put them into supplement. The IF images should have accompanied quantification. In general, the figures could use some work as they are difficult to understand as presented. Each figure should tell a precise part of the overall story and I feel like they are now a bit disconnected in message. Having them separate from the legends at the end of the manuscript does not help interpretation.
Response 3: Thank you for your suggestion, we have modified the figures and added qualifications to improve readability

Comment 4th
The appearance of the T stain looks like the cells are differentiating away from the main body of the aggregate. This is a potentially interesting characteristic. Was this observed only for cells staining brachyury or was this also observed for the endoderm populations?
Response 4: Thank you for your insightful question; indeed, this is a very interesting aspect that we are currently focusing on. We have already made some fascinating findings. However, we are not currently ready to disclose these results. Hopefully, we will publish these findings soon.

Comment 5 th
Figure 5 E –the label up-soft down hard is difficult to understand
Response 5: We have improved the figure.

Comment 6th
Figure 6 D –the axis label is cut off and it's difficult to follow what this graph is showing
Response 6: Apologize for our oversight, we have corrected the figure.

Regarding the reviewer #2:

Comment 1st
The phrase "Biocompatible material that allows spatial control of the chemical and mechanical environment for hPSC culture has not yet been developed" should be removed from the abstracts. As I noted in my previous review, such biocompatible materials have been developed.

Response 1: We appreciate your comment. Taking your feedback into consideration, we have revised our abstract by removing the sentence 'Biocompatible material that allows spatial control of the chemical and mechanical environment for hPSC culture has not yet been developed.' Thank you for your valuable input.

Comment 2nd
Quantitative analyses and statistics are still required (as noted in previous review) for Fig. 4 and 5.

Response 2: In response to your suggestion, we have quantified Figure 5 to depict the positive cell ratio. The quantification details have been moved to the supplementary figure. Additionally, following the advice of Reviewer 1, we have reintegrated qPCR to main figure to enhance the storyline.

Regarding Figure 5, at 72 hours, cells expressing T undergo EMT, leading to active migration within the colony, resulting in a thickened structure. The thickened structure made direct cell counting challenging. Therefore, we opted to quantify the T-positive area based on the maximum projection of immunofluorescence. Although this is an indirect measure, we believe it provides significant quantification and hope it convincingly supports our findings.

Comment 3rd
For all statistical analyses, please include number of samples and biological replicates in the caption (and of course there should be at least two biological replicates)

Response 3: Thank you for your valuable comment. We have added these descriptions into all figures.

© 2024. Published by The Company of Biologists under the terms of the Creative Commons Attribution License (https://creativecommons.org/licenses/by/4.0/).

Comment 4th
Quantification of YAP should be of the nuclear: cytoplasmic ratio, not average intensity. Similarly, some quantification of b-catenin localization to cell border would be helpful.

Response 4: Thank you for your advice. Following your suggestion, we have reanalyzed our data, and we found a significant difference in YAP after ROCK inhibition. The lack of a significant change in YAP using mean intensity is likely attributed to the flattened and expanded morphology observed after ROCK inhibition.

Regarding β-catenin quantification, the thick structure and tight packing of cells within colonies, along with unequal distribution along the Z-axis and variability between cells, posed challenges on quantification of border itself. Due to these problems, we initially used multiple Z-sections to quantify an overall trend of β-catenin as clamed in the main text of paper, aiming to minimize potential human error in choosing quantification objects. We hope the substantial color difference between hard and soft regions in the heatmap can convincingly support our findings."

Third decision letter

MS ID#: DEVELOP/2023/201621

MS TITLE: Spatial cell fate manipulation of human pluripotent stem cells by controlling the microenvironment using photocurable hydrogel

AUTHORS: Zhe Wang, Akira Numada, Yusuke Oda, Fumi Wagai, Masatoshi Ohgushi, Koichiro Maki, Taiji Adachi, and Mototsugu Eiraku

I have now received all the referees reports on the above manuscript, and have reached a decision. The referees' comments are appended below, or you can access them online: please go to BenchPress and click on the 'Manuscripts with Decisions' queue in the Author Area.

The overall evaluation is positive and we would like to publish a revised manuscript in Development, provided that the referees' comments can be satisfactorily addressed. Please attend to all of the reviewers' comments in your revised manuscript and detail them in your point-by-point response. If you do not agree with any of their criticisms or suggestions explain clearly why this is so. If it would be helpful, you are welcome to contact us to discuss your revision in greater detail. Please send us a point-by-point response indicating your plans for addressing the referees' comments, and we will look over this and provide further guidance.

Reviewer 1

Advance summary and potential significance to field

This study uses a biomaterials system to explore pl;uripotency regulation and differentiation on account of matrix stiffness. This work has potential to contribute to the emerging field of synthetic embryology where biophysical and biochemical attributes of the microenvironment might be revealed.

Comments for the author

The authors have responded sufficiently to the previous specific queries, and the inclusion of the peptide 17 results supports the idea that disrupting nuclear YAP activity abrogates mesoderm specification. However, the flow of the story remains difficult to follow and the figures remain confusing. Each revision seems to have a complete shuffling of data from main text to supplement and back without clear rationale. I still find it very difficult to extract a clear message from this manuscript. The following points should be addressed:

© 2024. Published by The Company of Biologists under the terms of the Creative Commons Attribution License (https://creativecommons.org/licenses/by/4.0/).

1. In figure 3, it is not clear why only neural ectoderm and definitive endoderm included the soft gel condition while the mesoderm diff study only includes the hard gel. This should be justified in the results and discussion.

2. The authors describe the use of YAP inhibition via peptide 17 and refer to Fig6b but this is a diagram of the protocol. The data for YAP localisation and effect on T after treatment should be in a main figure.

3. I believe the final mechanism shown in Fig5 is reasonably supported by the data. However, it is not clear how these different signalling pathways coincide/relate

---

Third revision

Author response to reviewers' comments

Regarding the reviewer #1:
Comment 1st
In figure 3, it is not clear why only neural ectoderm and definitive endoderm included the soft gel condition while the mesoderm diff study only includes the hard gel. This should be justified in the results and discussion.
Response 1:
Thank you for your suggestions. In Fig. 3, our primary focus was initially on demonstrating that our hydrogel can be used for differentiation rather than discussing the effect of stiffness. In response to reviewer 2's suggestion of performing qPCR on soft gel for endoderm and mesoderm differentiation during first round revision, and as RNA-seq suggested upregulation of endoderm-related genes, we concentrated on both endoderm and mesoderm, considering their shared origin. Furthermore, we directly tested early mesoderm on a patterned gel, as early mesoderm differentiation was shown to be affected by colony size. Bulk analysis methods like qPCR could lead to oversight of differences, given that both hard and soft gels are much softer compared to a dish. Using a patterned gel helped mitigate the effects caused by unequal cell distribution and provided a relatively homogeneous condition. We have rewritten this part to make the storyline clearer.

Comment 2nd
The authors describe the use of YAP inhibition via peptide 17 and refer to Fig6b but this is a diagram of the protocol. The data for YAP localisation and effect on T after treatment should be in a main figure.

Response 2: It appears that Fig. 5 and Fig. 6 were reversed in the previous submission. We sincerely apologize for the confusion and have corrected this mistake. Regarding Peptide17, since it acts by interrupting the interaction of YAP/TEAD, we did not test its effect on YAP localization.

Comment 3rd
I believe the final mechanism shown in Fig5 is reasonably supported by the data. However, it is not clear how these different signaling pathways coincide/relate.

Response 3: Thank you for your valuable suggestion. We acknowledge that exploring the relationship between YAP and Wnt signaling is an intriguing aspect. While many researchers have delved into this area and proposed hierarchies, we have encountered challenges in finding a definitive conclusion. However, we believe that investigating this hierarchy might deviate from the primary focus of our paper, which is centered around introducing a novel technique. Moreover, delving into the intricate details of the YAP/Wnt interaction within the constraints of our current research length presents a considerable challenge. We appreciate your understanding in this matter. Additionally, we have included a section in the discussion addressing the YAP and Wnt hierarchy.

© 2024. Published by The Company of Biologists under the terms of the Creative Commons Attribution License (https://creativecommons.org/licenses/by/4.0/).

Fourth decision letter

MS ID#: DEVELOP/2023/201621

MS TITLE: Spatial cell fate manipulation of human pluripotent stem cells by controlling the microenvironment using photocurable hydrogel

AUTHORS: Zhe Wang, Akira Numada, Yusuke Oda, Fumi Wagai, Masatoshi Ohgushi, Koichiro Maki, Taiji Adachi, and Mototsugu Eiraku
ARTICLE TYPE: Techniques and Resources Article

I am happy to tell you that your manuscript has been accepted for publication in Development, pending our standard ethics checks.

Reviewer 1

Advance summary and potential significance to field

This paper describes a photocurable hydrogel system for pluripotent stem cell culture. New systems to direct embryogenesis and stem cell differentiation have potential to advance the field of developmental biology. therefore, this tool could prove useful in studying these systems.

Comments for the author

The authors have satisfactorily responded to my concerns.

© 2024. Published by The Company of Biologists under the terms of the Creative Commons Attribution License (https://creativecommons.org/licenses/by/4.0/).
